# Individuals with controlled hypertension show endothelial integrity following a bout of moderate-intensity exercise: randomized clinical trial

**DOI:** 10.1038/s41598-021-87990-6

**Published:** 2021-04-20

**Authors:** Gustavo Waclawovsky, Liliana F. C. Boll, Bruna Eibel, Ana Paula Alegretti, Fabiane Spagnol, Juliana De Paoli, Simone Wajner, Rafael A. Marschner, Maximiliano I. Schaun, Alexandre Machado Lehnen

**Affiliations:** 1 Institute of Cardiology of Rio Grande do Sul/University Foundation of Cardiology, Av. Princesa Isabel, 395 Santana, Porto Alegre, RS 90620-001 Brazil; 2 Clinical Pathology Laboratory, Hospital de Clínicas de Porto Alegre, Universidade Federal do Rio Grande do Sul, Porto Alegre, RS Brazil; 3 Thyroid Section, Endocrine Division, Hospital de Clínicas de Porto Alegre, Universidade Federal do Rio Grande do Sul, Porto Alegre, RS Brazil

**Keywords:** Hypertension, Vascular diseases, Quality of life

## Abstract

To examine the acute effects of aerobic exercise (AE), resistance exercise (RE) or combined exercise (CE) on flow-mediated dilation (FMD), progenitor cells (PCs), endothelial progenitor cells (EPCs), oxidative stress markers and endothelial-cell derived microvesicles (EMVs) in patients with hypertension. This is a randomized, parallel-group clinical trial involving an intervention of one session of three different modalities of exercise. Thirty-three males (43 ± 2y) were randomly divided into three groups: a session of AE (n = 11, 40 min, cycle ergometer, 60% HRR); a session of RE (n = 11, 40 min, 4 × 12 lower limb repetitions, 60% 1-RM); or a session of CE (n = 11, 20-min RE + 20-min AE). FMD was assessed 10 min before and 10, 40 and 70 min post-intervention. Blood samples were collected at the same time points (except 40 min). FMD were similar in all groups and from baseline (within each group) after a single exercise bout (AE, RE or CE). At 70 min, RE group showed higher levels of PCs compared to the AE (81%) and CE group (60%). PC levels were reduced from baseline in all groups (AE: 32%, *p* = 0.037; RE: 15%, *p* = 0.003; CE: 17%, *p* = 0.048). The levels of EPCs, EMVs and oxidative stress were unchanged. There were no acute effects of moderate-intensity exercise on FMD, EPCs, EMVs and oxidative stress, but PCs decreased regardless of the exercise modality. Individuals with controlled hypertension do not seem to have impaired vascular function in response to a single exercise bout.

## Introduction

Systemic arterial hypertension is a multifactorial clinical condition characterized by sustained high blood pressure (BP) levels^1^. An increase of 20 mmHg in systolic blood pressure (SBP) has been associated with a two-fold increased risk of death from ischemic heart disease due to vascular disease^2^. It has been proposed that exacerbated sympathetic activity plays an important role in the development and maintenance of hypertension^3^.

The endothelium plays a central role in the modulation of angiogenesis, inflammatory response, regulation of vascular tone and peripheral vascular resistance^4^. It is well known that cardiovascular events are directly associated with impaired endothelial function^5^ characterized by decreased production and bioavailability of nitric oxide (NO) and/or insufficient vasomotor response. Flow-mediated dilation (FMD) is an important non-invasive method for measuring vascular function^6^, and FMD results from a single exercise session may predict adaptive training changes^7^.

Endothelial-cell derived microvesicles (EMVs) are located in the membrane of endothelial cells and are released after cellular activation or apoptosis of these cells. Thus, EMVs are biomarkers of endothelial damage by increased circulating EMVs. In the long term, aerobic exercise training may decrease resting levels of EMVs in healthy individuals, which may reflect reduced vascular injury^8^. In an acute setting (i.e., short-term after a single exercise session), circulating EMV levels increased 90 and 120 min post-exercise in healthy individuals^9^ and EMV levels remain unchanged (10–30 and 60 min) after a session of resistance exercise (RE)^10^.

It is well established that 3% of bone marrow cells expressing CD34 (progenitor cells, PCs) can reconstitute long-term multilineage hematopoiesis. CD34 + cells can also be found in peripheral blood of healthy individuals though they are extremely rare (around 0.01–0.05% of total nucleated cells)^11^. Evidences demonstrate that CD34 + cells isolated from peripheral blood are capable of forming colonies of endothelial cells^12^. Thus, PCs play a role in the maintenance and increase of bone marrow-derived endothelial progenitor cells (EPCs). EPCs migrate to peripheral blood and differentiate into mature endothelial cells and contribute to endothelial recovery through stimulating vascular factors including granulocyte-colony stimulating factor (G-CSF)^13^ and vascular endothelial growth factor (VEGF)^10^, induction of hypoxia-inducible factor-1 (HIF-1) and increase in NO levels^14^. EPCs have been associated with cardiovascular risk factors^15^, FMD^14^, and mortality from cardiovascular diseases^16^. EPC number and function are reduced among participants with hypertension when compared to healthy ones^17,18^ and low circulating levels of EPCs may contribute to endothelial dysfunction and increased risk of atherosclerosis in this population^19,20^. Evidence suggests that aerobic exercise (AE) training improves FMD in patients with hypertension^21^, increases the levels of EPCs in individuals with chronic heart failure^22^, and improves FMD immediately after and 24 h post-session in individuals with metabolic syndrome^23^. Increased circulating EPCs have been observed 20 min post-exercise in healthy individuals^14^. A single RE session at low to moderate intensity has been shown to reduce FMD 10–30 and 60 min post-exercise^24^ and to increase the levels of EPCs 120 min post-exercise in healthy individuals^10^.

Despite FMD improvement in response to AE among patients with hypertension, the acute effects of a single exercise bout on FMD in this population are not yet clear. To the best of our knowledge, a good number of studies that investigated the effects of one exercise session on FMD and EPCs did not include volunteers with hypertension and the scant evidence available is limited to AE^21,25^. Therefore, we aimed to examine the acute effects of a single session of AE, RE or CE on FMD and, secondarily, on the levels of PCs, EPCs and EMVs, and oxidative stress parameters in participants with hypertension. We postulated that all exercise modalities here studied can effectively improve FMD and increase circulating levels of EPCs, EMVs and oxidative stress parameters in individuals with hypertension, being more pronounced with AE.

## Results

**Figure 1:**
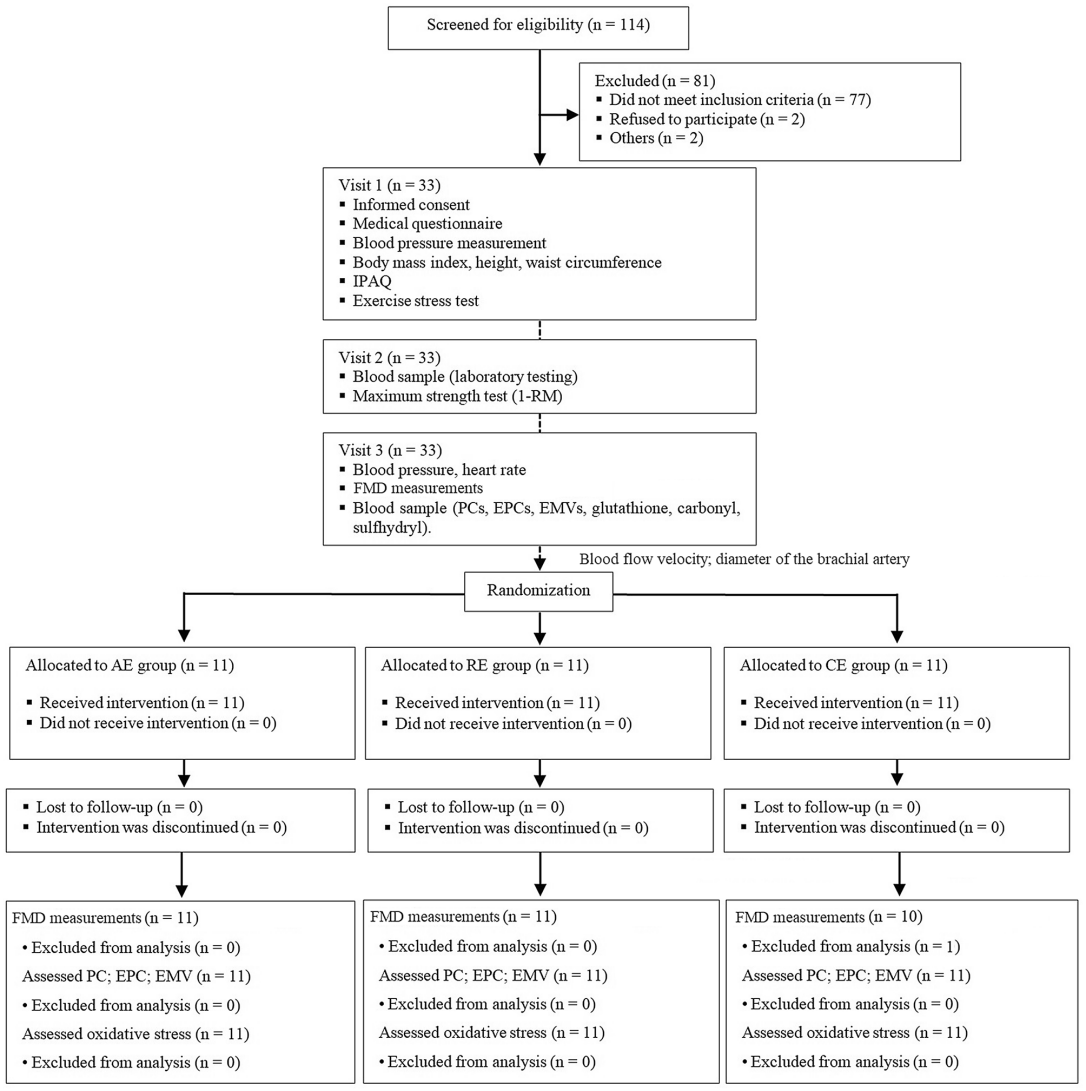
Study design. IPAQ, International Questionnaire of Physical Activity; FMD, flow-mediated dilation; PCs, progenitor cells, EPCs, endothelial progenitor cells, EMVs, endothelial microvesicles; AE, aerobic exercise session; RE, resistance exercise session; CE, combined exercise session.

A total of 114 patients with hypertension receiving care at IC outpatient clinic were eligible to participate in the study from August 2016 to May 2018 (recruitment and follow-up). Of these, 81 were excluded and 33 were recruited and invited to participate (Fig. 1). Table 1 shows the characteristics of the study participants. Mean time from diagnosis of hypertension was 10 years with SBP 134.8 ± 9.8 mmHg and DBP 81.4 ± 8.5 mmHg. Age, BMI, waist circumference, SBP/DBP, aerobic capacity determined using the Bruce test protocol and maximum strength were similar in all groups. Triglyceride levels were higher in the CE group (~ 67%) compared to the AE group and HDL levels were lower in the CE group (~ 22%) compared to AE and RE groups. No difference was found in the level of physical activity evaluated by IPAQ METs among the groups (*p* = 0.265): 18 participants were considered insufficiently active (AE 7; RE 4; CE 7), 11 sufficiently active (AE 3; RE 5; CE 3), and four very active (AE 1; RE 2; CE 1).

**Table 1:** Characteristics of participants undergoing three different exercise interventions.

Variables	AE (n = 11)	RE (n = 11)	CE (n = 11)	*p*-value
Age (years)	45.5 ± 10.0	43.6 ± 15.9	45.4 ± 7.2	0.914
Duration of HT (months) (95%CI)	96 (19–202)	96 (40–214)	60 (18–187)	0.905
BMI (kg/m^2^)	29.0 ± 3.6	30.5 ± 3.3	30.6 ± 4.4	0.491
Waist circumference (cm)	96 ± 13	103 ± 11	100 ± 13	0.455
SBP (mmHg)	132 ± 11	137 ± 10	137 ± 9	0.437
DBP (mmHg)	80 ± 6	83 ± 10	81 ± 10	0.610
Fasting blood glucose (mg/dL)	97.7 ± 12.2	102.8 ± 13.3	100.1 ± 18.8	0.773
HbA1c (%)	5.3 ± 0.4	5.5 ± 0.4	5.7 ± 0.8	0.337
GFR (mL/min/1.73m^2^)	91.2 ± 14.3	99.7 ± 21.5	91.2 ± 18.3	0.458
Triglycerides (mg/dL)	123.8 ± 40.2	144.0 ± 48.2	206.6 ± 111.2*	0.040
Total cholesterol (mg/dL)	192.0 ± 33.2	217.0 ± 73.3	197.1 ± 57.8	0.562
HDL cholesterol (mg/dL)	49.0 ± 12.4	45.4 ± 11.2	36.8 ± 7.4*^†^	0.032
LDL cholesterol (mg/dL)	118.2 ± 27.9	111.9 ± 42.4	100.8 ± 66.5	0.697
VO_2_peak (mL/kg/min)**	47.4 ± 9.7	44.3 ± 10.3	42.8 ± 9.5	0.552
METs/week—IPAQ	1154 ± 1224	1986 ± 1866	1067 ± 1070	0.265
1-RM test for knee extension (kg)	71.8 ± 8.6	64.6 ± 15.0	66.0 ± 23.2	0.594
1-RM test for knee flexion (kg)	74.9 ± 14.0	70.8 ± 14.6	72.9 ± 16.7	0.826
1-RM test for leg press (kg)	110.3 ± 13.6	107.3 ± 11.3	98.6 ± 12.1	0.097
1-RM test for plantar flexion (kg)	105.9 ± 11.2	107.4 ± 11.3	101.3 ± 8.9	0.387
**Antihypertensive drugs**				
Diuretics (n)	2	1	0	–
Additional diuretics in the drug schedule (n)	6	6	7	–
ACEI/ARBs (n)	9	10	10	–
Calcium channel blockers (n)	0	2	4	–
Betablockers (n)	0	0	1	–

Values are expressed as mean ± standard deviation. Duration of hypertension is expressed as median (95% CI). AE: aerobic exercise session; RE: resistance exercise session; CE: combined exercise session; GFR (glomerular filtration rate) was calculated by The Modification of Diet in Renal Disease (MDRD) Study equation; BMI: body mass index; SBP: systolic blood pressure; DBP: diastolic blood pressure; HbA1c: glycated hemoglobin.

**VO_2_peak: peak oxygen consumption predicted by the Bruce protocol; METs: metabolic equivalents; ACEI: angiotensin converting enzyme inhibitors; ARBs: angiotensin II receptor antagonists. ANOVA one-way with Bonferroni post-hoc test.

*p < 0.05 versus AE.

^†^*p* < 0.05 versus RE.

### Blood flow response and flow-mediated dilation

**Figure 2:**
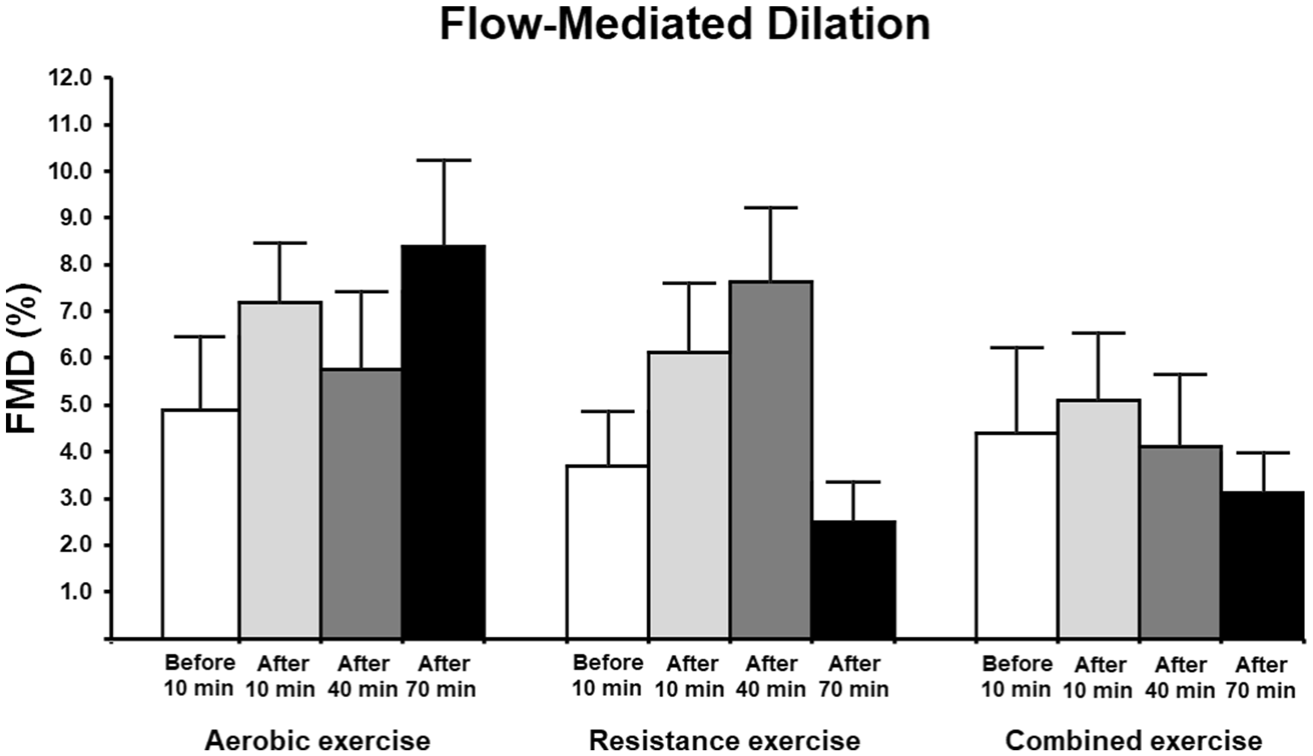
Flow-mediated dilation of the brachial artery at baseline and after the exercise intervention in participants with hypertension. AE: aerobic exercise session (n = 11); RE: resistance exercise session (n = 11); and CE: combined exercise session (n = 10). Values are expressed as mean ± standard error. GEE with Bonferroni post-hoc test was used (*p* < 0.05) and no difference was observed within each group and among the groups; *p* (group) = 0.114, *p* (time) = 0.263, and *p* (interaction) = 0.157.

Figure 2 shows FMD assessment. There were no changes in FMD (%) in response to a single exercise session (intervention)—*p* (interaction) = 0.157; similarly, there was no change in FMD assessment with the allometric scaling procedure (Table 2). Additionally, the analysis of the effect size by exercise modality and time showed that the largest effect size of AE was at 70 min post-session (0.62—medium effect) while the largest effect size of RE was at 40 min post-session (0.85—large effect). Interestingly, non-relevant effect size (0.13) was observed on FMD of CE shortly after the end of the session. Individual data points can be found in the supplementary material.

**Table 2:** Diameter of the brachial artery, time to peak flow-mediated dilation, shear rate and blood pressure in participants with hypertension undergoing three different exercise interventions.

	10 min before exercise	10 min after exercise	40 min after exercise	70 min after exercise	*p*-value (group)	*p*-value (time)	*p*-value (interaction)
**Baseline diameter (mm)**					0.820	0.233	0.222
AE	4.6 ± 0.1	4.6 ± 0.2	4.7 ± 0.1	4.5 ± 0.1			
RE	4.6 ± 0.2	4.6 ± 0.2	4.7 ± 0.1	4.6 ± 0.2			
CE	4.4 ± 0.2	4.5 ± 0.3	4.5 ± 0.2	4.5 ± 0.2			
**Peak diameter (mm)**—**FMD**					0.676	0.169	0.695
AE	4.9 ± 0.1	4.9 ± 0.1	4.9 ± 0.1	4.9 ± 0.1			
RE	5.0 ± 0.2	4.9 ± 0.2	5.0 ± 0.2	4.8 ± 0.2			
CE	4.7 ± 0.3	4.7 ± 0.3	4.7 ± 0.2	4.6 ± 0.3			
**Peak diameter (allometric scale)**—**FMD**					0.080	0.451	0.244
AE	1.25 ± 0.07	1.27 ± 0.04	1.24 ± 0.07	1.28 ± 0.06			
RE	1.26 ± 0.11	1.25 ± 0.05	1.25 ± 0.09	1.21 ± 0.03			
CE	1.27 ± 0.14	1.23 ± 0.05	1.23 ± 0.05	1.21 ± 0.04			
**Time to peak (s)**—**FMD**					0.005	0.105	0.330
AE	58.1 ± 10.4	63.5 ± 12.1	70.7 ± 15.6	57.8 ± 14.9			
RE	52.6 ± 9.3	74.5 ± 7.9	89.7 ± 16.2	95.4 ± 15.9			
CE	86.4 ± 13.1	82.5 ± 13.9	97.7 ± 12.4	65.0 ± 8.0			
**Mean resting SR-AUC (s, 10**^**3**^**)**					0.007	< 0.001	0.258
AE	8.6 ± 1.6	12.6 ± 1.4	9.1 ± 1.0	5.9 ± 1.1			
RE	10.5 ± 1.54	14.1 ± 1.8	8.8 ± 1.6	6.6 ± 1.4			
CE	9.9 ± 1.1	17.9 ± 1.5	11.3 ± 1.5	10.5 ± 1.4			
**Mean SR-AUC (s, 10**^**3**^**)**—**FMD**					0.031	0.051	0.126
AE	15.7 ± 2.7	23.0 ± 3.7	25.2 ± 5.8	14.1 ± 2.9			
RE	16.2 ± 2.4	23.5 ± 3.4	21.2 ± 3.3	29.4 ± 5.1			
CE	26.4 ± 5.6	27.7 ± 6.6	34.0 ± 4.1	30.7 ± 4.6			
**Δ diameter adjusted by SR-AUC (mm/s, 10**^**3**^**)**					0.140	0.717	0.159
AE	0.015 ± 0.002	0.023 ± 0.009	0.011 ± 0.009	0.047 ± 0.018			
RE	0.026 ± 0.011	0.104 ± 0.081	0.027 ± 0.007	0.009 ± 0.004			
CE	0.017 ± 0.007	0.006 ± 0.004	0.010 ± 0.004	0.008 ± 0.002			
**SBP (mmHg)**					0.304	< 0.001	0.437
AE	134.8 ± 5.4	134.5 ± 4.7	128.2 ± 4.9	130.7 ± 5.8			
RE	139.3 ± 3.0	145.4 ± 3.3*†ǂ	136.0 ± 3.2	138.5 ± 3.6			
CE	138.9 ± 3.6	144.1 ± 3.5†ǂ	131.3 ± 2.3*	137.0 ± 2.7			
**DBP (mmHg)**					0.982	0.002	0.405
AE	84.2 ± 2.7	84.5 ± 2.9	78.2 ± 2.6	81.6 ± 3.3			
RE	82.7 ± 4.1	82.0 ± 4.1	80.9 ± 3.4	81.6 ± 3.8			
CE	82.0 ± 2.7	80.2 ± 2.7	80.2 ± 2.4	82.5 ± 3.5			

Data described as mean ± standard error. AE: aerobic exercise session (n = 11); RE: resistance exercise session (n = 11); CE: combined exercise session (n = 10); Allometric scaling is given by [peak diameter/(baseline diameter)^0,89^]. Mean SR-AUC: area under the curve of mean resting shear rate (SR) to peak FMD; Δ diameter adjusted by SR-AUC is given by [(peak diameter minus baseline diameter) divided by (mean SR-AUC minus mean resting SR-AUC)] for each individual data. SBP: systolic blood pressure in supine position; DBP: diastolic blood pressure in supine position. GEE with Bonferroni post-hoc test was used.

*p < 0.05 versus 10 min before exercise.

^†^*p* < 0.05 versus 40 min after exercise.

ǂ*p* < 0.05 versus 70 min after exercise.

FMD changes were also inversely correlated with baseline values at 10 and 40 min post-intervention in the AE group (r = − 0.785, *p* = 0.007, r = − 0.836, *p* = 0.001) and at 10, 40 and 70 min post-intervention in the RE group (r = − 0.860, *p* = 0.003, r = − 0.906, *p* < 0.001, r = − 0.976, *p* < 0.001) and in the CE group (r = − 0.959, *p* < 0.001, r = − 0.960, 0.001, r = − 0.981, *p* < 0.001). Table 2 shows FMD measurements including diameter of the brachial artery, time to peak flow-mediated dilation, shear rate and blood pressure.

**Figure 3:**
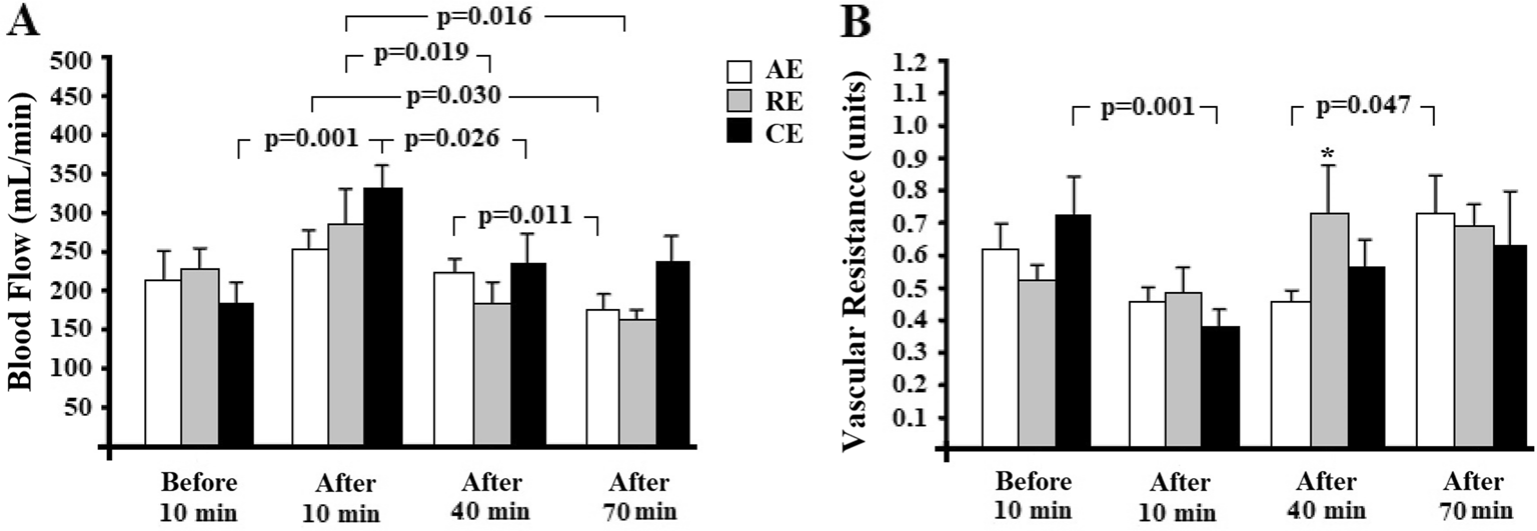
Blood flow response (**A**), vascular resistance (**B**) of the arm at baseline and after exercise intervention in participants with hypertension. AE: aerobic exercise session (n = 11); RE: resistance exercise session (n = 11); CE: combined exercise session (n = 10). Values are expressed as mean ± standard error. GEE with Bonferroni post-hoc test was used and considered differences for *p* < 0.05. Panel **A**: *p* (group) = 0.543, *p* (time) < 0.001, and *p* (interaction) < 0.001; Panel **B**: *p* (group) = 0.890, *p* (time) < 0.001, and *p* (interaction) = 0.024; * *versus* AE at the same time point.

Figure 3 shows blood flow response (Panel A) and peripheral vascular resistance (Panel B) in the left arm at baseline and after exercise intervention (a single bout of AE, RE or CE). Blood flow was similar among the groups at the time points studied. However, blood flow values ranged within each group. All exercise interventions reduced blood flow 10 min post-exercise until 70 min post-intervention (*p*-values are shown in Fig. 3A). Interestingly, there was no difference in the resting diameter of the brachial artery among the groups at the time points studied and no difference in baseline diameters within each group—Table 2. Higher peripheral vascular resistance values (Fig. 3B) were observed at 40 min post-intervention in the RE compared to the AE group (*p* = 0.022). Within each group, peripheral vascular resistance reduced in the CE group at 10 min post-exercise (*p* = 0.001) from baseline and in the AE at 40 min versus 70 min after intervention (*p* = 0.047).

### Progenitor cells, endothelial progenitor cells and microvesicles

**Figure 4:**
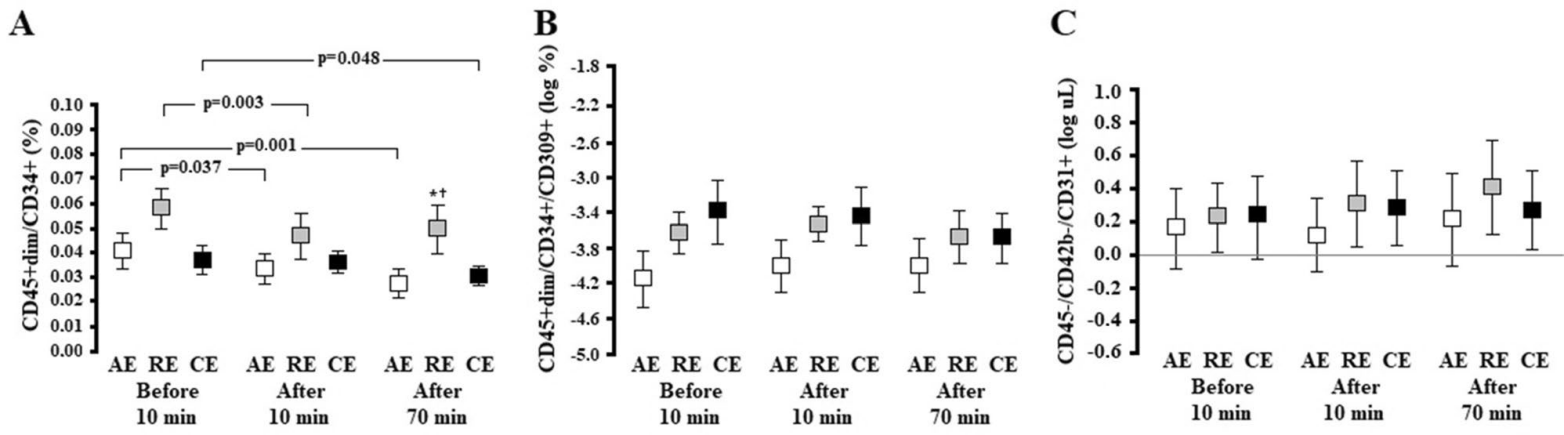
Response of PCs: CD45 + dim/CD34 + (**A**), EPCs—CD45 + dim/CD34+/CD309 + (**B**) and EMVs—CD45−/CD42b−/CD31 + (**C**) at baseline and after exercise interventions in participants with hypertension. AE: aerobic exercise session (n = 11); RE: resistance exercise session (n = 11); CE: combined exercise session (n = 11). Values are expressed as mean ± standard error. GEE followed by Bonferroni post-hoc test was used and considered differences for *p* < 0.05. Panel **A**: *p* (group) = 0.034, *p* (time) < 0.001, and *p* (interaction) = 0.025; Panel **B**: *p* (group) = 0.033, *p* (time) = 0.186, and *p* (interaction) = 0.111; Panel **C**: *p* (group) = 0.638, *p* (time) = 0.149, and *p* (interaction) = 0.840; * versus AE at the same time point; † versus CE at the same time point.

The RE group showed higher PC levels at 70 min post-intervention when compared to the AE (*p* = 0.005) and CE groups (*p* = 0.009) (Fig. 4A). Within each group, PC levels were lower at 10 min post-intervention in the AE (*p* = 0.037) and RE groups (*p* = 0.003). These levels were reduced only at 70 min post-intervention in the CE group (*p* = 0.048). Finally, when analyzing the effect of time, exercise modality and interaction between these factors, AE, RE and CE sessions did not promote changes in the levels of EPCs (Fig. 4B) and circulating EMVs (Fig. 4C).

### Parameters of oxidative stress

Intragroup analyses showed no differences in sulfhydryl, GSH and GSSG content. The exercise intervention did not promote any change in GSH/GSSG ratio and carbonyl content. There were no changes in the levels of these parameters at the end of each exercise session. Table 3 details the parameters of oxidative stress by intervention group and time point studied.

**Table 3:** Comparison of oxidative stress parameters in patients with hypertension undergoing three different exercise interventions.

	10 min before exercise	10 min after exercise	70 min after exercise	*p*-value (group)	*p*-value (time)	*p*-value (interaction)
**Sulfhydryl (nmol)**						
AE	0.130 ± 0.006	0.133 ± 0.011	0.127 ± 0.011	0.333	0.223	0.819
RE	0.124 ± 0.015	0.139 ± 0.017	0.128 ± 0.006			
CE	0.211 ± 0.067	0.220 ± 0.061	0.153 ± 0.023			
**GSH (nmol)**				0.001	0.710	0.603
AE	11.528 ± 0.765	10.720 ± 0.838	11.011 ± 1.139			
RE	7.543 ± 0.608	8.162 ± 0.474	8.183 ± 0.778			
CE	8.171 ± 0.821	7.585 ± 0.691	8.770 ± 1.154			
**GSSG (nmol)**				< 0.001	0.903	0.236
AE	0.604 ± 0.013	0.616 ± 0.082	0.608 ± 0.018			
RE	0.606 ± 0.023	0.578 ± 0.006	0.606 ± 0.024			
CE	0.509 ± 0.025	0.514 ± 0.023	0.497 ± 0.025			
**GSH/GSSG ratio**				< 0.001	0.548	0.305
AE	19.041 ± 1.215	17.496 ± 1.373	18.552 ± 2.160			
RE	12.513 ± 1.048	14.154 ± 0.871	13.602 ± 1.258			
CE	15.346 ± 2.489	15.129 ± 1.482	18.673 ± 2.932			
**Carbonyl (nmol)**				0.840	0.660	0.468
AE	0.432 ± 0.052	0.597 ± 0.102	0.583 ± 0.094			
RE	0.568 ± 0.075	0.569 ± 0.060	0.570 ± 0.105			
CE	0.592 ± 0.068	0.576 ± 0.117	0.571 ± 0.081			

Data described as mean ± standard error. AE: aerobic exercise session (n = 11); RE: resistance exercise session (n = 11); CE: combined exercise session (n = 11). GSH, reduced glutathione; GSSG, oxidized glutathione; GSH/GSSG, ratio between reduced and oxidized glutathione. GEE with Bonferroni post-hoc test was used (*p* < 0.05).

## Discussion

We investigated the effects of AE, RE and CE on FMD, PCs, EPCs, EMVs and oxidative stress parameters in individuals with hypertension. The main finding of our study was that a single moderate-intensity exercise session did not change FMD of the brachial artery (untrained limb), but reduced the levels of PCs from baseline, within each group. Moreover, our exercise intervention did not promote any changes in the levels of EPCs, oxidative stress parameters and EMVs. Since there was no evidence of induction of endothelial cell injury as the redox state and circulating EMV levels remained unchanged, this finding may in part indicate that a single session of AE, RE or CE and the times studied were safe and maintained vascular integrity in this population of individuals with hypertension with reduced endothelial regenerative capacity^19^. To the best of our knowledge, other authors have conducted similar studies, but this is the first study to assess the effects of AE, RE and CE on vascular response associated with the levels of PCs, EPCs and circulating EMVs in a group of individuals with hypertension.

Improvement (or not) of upper limb endothelial function in response to a single exercise bout has been investigated with conflicting results reported. Some studies have demonstrated improved endothelial function after a session of AE^26–28^, while others did not find this same benefit^29–31^. Dawson et al.^7^ reported reduced FMD in healthy individuals 30 min after a session of moderate-intensity AE. Exercise intensity may have played a direct role and explain these conflicting results^32^. Hence, greater intensities may induce more pronounced reductions in FMD post-exercise while mild or moderate exercise intensities may not induce this same effect^33,34^.

Low- to moderate-intensity RE of lower limbs has been described to reduce FMD in healthy individuals at 10, 30 and 60 min post-intervention^24^. Interestingly, these authors reported that FMD remains at baseline levels with incremental exercise intensity and less repetitions^24^. We chose to examine moderate-intensity exercise in this study because it is widely recommended for antihypertensive management^35^. Green et al.^36^ have demonstrated incremental vasodilation of the brachial artery during lower-limb AE with higher exercise intensities in healthy individuals. Yet, Birk et al.^33^ have reported an inverse relationship between exercise intensity and FMD response immediately after AE^33^. It is not yet clear how exercise intensity influences the immediate post-exercise FMD responses. Different factors may play a role including higher blood pressure levels (AE: higher intensities; RE: lower intensities and more repetitions) with continuously increasing blood flow during an exercise bout resulting in a decrease in post-ischemia shear stress induced by cuff deflation and/or NO substrate limitation due to sustained increases in shear stress^37^. However, these mechanisms need further investigation.

Vasoconstriction or unchanged FMD in response to a single exercise bout can also be partly explained by an increase in the diameter of the brachial artery from baseline after workout^7^. In healthy individuals, an increase in baseline diameter of the brachial artery after a workout session is apparently similar for AE and CE and lasts up to an hour post-exercise^37^. We found no changes in baseline diameter data in the participants of the three intervention groups at any of the time points studied, a finding that is supported by other studies^23,24^ and rules out a potential baseline diameter effect on our FMD findings. Thus, unchanged FMD values in the group x time interaction analysis could be due to the absence of a control group rather than due to physiological causes or inadequate statistical power. In particular, we believe that FMD data at 70 min post-exercise session are not statistically significant because the one-third difference in values at 40 min is "smoothed" in the GEE analysis (group x time interaction). However, when considering the physiological significance of FMD finding rather than its statistical significance, the changes in vascular function are quite large. A 1% difference in FMD is generally considered clinically significant and is associated with an 8–13% difference in cardiovascular disease risk^38^. In our results, we reported changes in FMD of at least 3.5% within each exercise modality. Thus, from a time perspective (within-group), and considering the magnitude of FMD changes and their clinical importance (FMD ≥ 1%), our results show cardiovascular protective effects in response to a single session of AE and RE (up to 40 min), but not to a single session of CE. Interestingly, the magnitude of FMD response was greater 70 min after the exercise for AE compared to 40 min after the exercise for RE. This time variation in FMD response to one exercise session may be related to the stimulus profile. One hypothesis is that AE induces prolonged steady increases in shear stress while RE induces intermittent though transient increases (similar to ischemia–reperfusion) with NO synthesis and consequent vasodilation occurring earlier^37,38^. Another hypothesis is that SBP is higher 10 min after the RE session (Table 2) and may be associated with higher acute circulating levels of endothelin-1 (the most potent endogenous vasoconstrictor)^39^, which in turn may lead to a compensatory wave effect in the FMD response 40 min after the RE session. On the other hand, FMD changes to AE follow a more linear growth pattern.

Evidence shows that variations in shear stress induced by AE and RE may have an impact on the release of endothelial NO, which points to the importance of different responses to these specific exercise modalities for vascular adaptation^40^. In this sense, similar FMD responses to AE, RE and CE (group x time interaction analysis) might suggest similar increases in blood flow (anterograde and/or retrograde) to the upper limbs leading to average shear stress comparable across the three exercise groups. However, these vascular parameters were not assessed during our exercise sessions, which precludes further interpretations. Current evidence suggests that, despite an adequate shear rate applied during AE at moderate intensities, vasoconstriction in response to sympathetic nervous system activation competes with endothelium-dependent vasodilator activity, especially in untrained limbs following one exercise bout^41^. This mechanism may have influenced our results.

Factors such as exercise-induced oxidative stress may lead to a reduction in NO bioavailability and inhibition of endothelium-dependent vasodilation^34,42^. Evidence in healthy individuals points to a dose-dependent effect between oxidative stress and exercise intensity and/or duration of an AE^34^ or RE session^43^. In elderly individuals with hypertension, an AE session was shown to increase lipid peroxidation due to increased production of reactive oxygen species at 30 min after moderate-intensity exercise, but the same was not seen for mild-intensity exercise^44^. Bearing in mind that AE, RE and CE intervention in our study consisted of moderate-intensity structured exercise sessions, it would be expected an acute response of increased oxidative stress induced by exercise and associated with endothelial damage. However, our results did not show such changes, which suggests other factors such as exercise-induced shear stress rate may play a role. Evidence shows FMD changes result from exercise-induced increases in shear stress^45^, which are intensity-dependent and affected by local vasodilatory mechanisms^41^.

To examine endothelial response to different exercise modalities, we measured vasomotor response by FMD and endothelial integrity through regenerative (EPCs) and harmful factors including oxidative stress and EMVs. In addition to FMD findings, we demonstrated that the levels of EPCs remained similar and PCs were reduced in participants with hypertension regardless of the exercise modality.

EPC and PC response to a similar single bout of AE and RE have been discussed by our elsewhere^46^. Both exercise modalities did not improve EPC and PC levels in individuals with type 1 diabetes mellitus until 10 min post-intervention. In contrast, in healthy controls, EPCs were reduced after AE and increased after RE and PCs decreased in both exercise modalities (AE and RE)^46^. Our findings in participants with hypertension, even with the addition of CE, are in agreement with those reported for individuals with type 1 diabetes mellitus.

AE has shown to potentially increase circulating levels and function of EPCs in individuals with cardiovascular risk factors or even established heart disease^25^. However, the acute effect of one exercise session as well as the acute response on EPC and PC levels in patients with hypertension has not yet been explored. In healthy individuals, EPCs and PCs increased following a single 30-min high-intensity AE session until 20 min post-intervention, and this effect was directly dependent on NO levels^14^. It suggests that endothelial regenerative capacity depends on the release of NO to the peripheral circulation to induce mobilization of EPCs and PCs from bone marrow.

PCs play a role in the maintenance and increase of EPCs through stimulating factors including G-CSF^13^ and VEGF^10^, induction of HIF-1 and increase in NO levels^14^. Following AE and RE interventions similar to those used in volunteers with hypertension in our study, no changes on circulating peripheral blood PC levels were found 10 min post-exercise in individuals with type 1 diabetes mellitus^46^. On the other hand, PC levels decreased in healthy controls. Van Craenenbroeck et al.^47^ found increased EPC levels and unchanged PC levels after maximal cardiopulmonary exercise testing in healthy subjects. Although it remains highly speculative, Van Craenenbroeck et al.^16^ suggested at that time that the maintenance of circulating PC levels post-exercise session likely reflected a balance between EPCs in the bone marrow niche released into circulation and their rate of utilization after release for endothelial recovery.

The mechanisms postulated for PC mobilization involves the activation of matrix metallopeptidase 9 (MMP-9) via a signaling pathway of nitric oxide synthase/NO/cyclic guanosine monophosphate in the bone marrow^48^. With animal experimentation, Aleksinskaya et al.^48^ have demonstrated that hypertension decreases NO levels in the bone marrow, which negatively affects the release of PCs into circulation. Given that shear stress during an exercise bout (a stimulus for NO release) is different in AE and RE^40^, we hypothesized that circulating NO levels would be different following each intervention of the study (AE, RE and CE). However, because of potentially decreased NO levels in the bone marrow, PC release may be reduced, which could partly explain different PC levels found post-intervention in our study. However, we did not assess either shear stress during exercise or NO markers in our study so we cannot make any inferences. Thus, we believe that the presence and the magnitude of the events described above may have caused the reduction in PC levels and different PC levels found post-exercise in volunteers with hypertension.

SBP is increased during RE in patients with hypertension when compared to normotensive individuals^49^. Since BP variation and/or sustained high BP levels occur during exercise, either aerobic or resistance, it would be expected an increase in endothelial injury^50^. Different BP stimuli may lead to varying degrees of endothelial damage, with a modality-specific response, through the action of EPCs as previously suggested^46^. However, we did not find endothelial damage by circulating EMVs following an exercise session in our study, which may in part explain unchanged circulating levels of EPCs.

Some limitations of this study should be noted. First, the study did not include a control group (no exercise intervention). We defined one aerobic exercise session as the gold standard modality for individuals with hypertension (as recommended in many guidelines) and compare RE and CE to this first-line strategy. Second, the sample size per intervention group was relatively small. We calculated the sample size per group for two intervention groups and then added a third group based on this calculation (before randomization). There was a slight decrease in the statistical power mostly because we used a different statistical procedure. Since our study did not include a control group and our sample size was small hypothesis testing was not expected to be significant for variables with low magnitude of change or transient effect. Thus, the statistical power calculated for FMD (clinical outcome) in the group x time interaction analysis was not adequate. However, our findings of FMD changes are clinically meaningful, in particular for AE and RE. To further support our results, we showed the effect size calculation (Cohen's D). Another limitation is that our sample consisted of male volunteers only. This was because menstrual cycle phases may interfere with the levels of EPCs and EMVs^51,52^. A larger sample would be required to show potential variations in menstrual cycle phase and its role as a co-factor. Yet, it would have increased project costs.

In conclusion, we found that FMD and circulating EPCs levels remained unchanged up to 70 min after the intervention (40 min of moderate-intensity AE, RE and CE). However, a reduction in PCs levels may occur regardless of the exercise modality and the magnitude of the response seems dependent on the aerobic component of the exercise session. Unchanged levels of parameters involved with oxidative stress and endothelial injury, marked by circulating EMVs, show integrity of the vascular bed despite increased BP levels following different modalities of moderate-intensity exercise.

## Methods

We conducted a randomized clinical trial following the Consolidated Standards of Reporting Trials (CONSORT) guidelines^53^ as well as the principles of the Declaration of Helsinki. This study was approved by the local institutional review board (Research Ethics Committee of Institute of Cardiology of Rio Grande do Sul/University Foundation of Cardiology, Porto Alegre, RS, Brazil; ID number: 5106/15; Date: 19/05/2015) and registered at www.clincaltrials.gov (ID number NCT02937922; Date: 17/10/2016).

### Study participants

Our sample consisted of 33 male adults with essential hypertension receiving outpatient care at the public clinic of Institute of Cardiology of Rio Grande do Sul (ICFUC) from August 2016 to May 2018 (recruitment and follow-up). The inclusion criteria were being an adult male aged 20–60 years with hypertension on continuous use of antihypertensive medication who did not engage in regular physical exercise (≥ 2 sessions per week). The exclusion criteria were diabetes mellitus; chronic renal failure; body mass index (BMI) ≥ 35 kg/m^2^; coronary artery disease; heart failure; statin use; any lower limb injury; and being a smoker. Additionally, as the menstrual cycle may interfere with the levels of EPCs and EMVs^51,52^ we chose not to include women in this study.

The calculation of the sample size was based on data from Feairheller et al.^8^ that evaluated FMD in 26 sedentary patients (with normal BP, prehypertension and hypertension) following an AE intervention. Assuming a standard deviation of 2.9 and 2.1, 3.7% difference (absolute) in FMD values (intra-group) and 90% power at a significance level of 0.05, a sample of 33 subjects was required (n = 11 per group).

Randomization of the study interventions (exercise groups) was performed with the use of a computer program (www.randomization.org) with a coded numeric distribution (1–2–3). Allocation concealment was guaranteed; the random allocation of participants was kept in an inaccessible place and researchers did not have a priori knowledge of the intervention assignment to each participant. A numeric sequence was generated by a researcher blinded to the study for the individuals meeting the inclusion criteria. The numeric sequence for randomization was kept confidential until the very beginning of the experiment. Given that comparative analyses of groups would involve baseline measurements, simple randomization was conducted for allocation to type of exercise session (1: aerobic exercise OR 2: resistance exercise OR 3: combined, aerobic + resistance exercise). All participants were blinded to group allocation (type of exercise) until the intervention day. The study evaluators were also blinded to the participants’ group allocation to minimize potential measurement biases.

### Visit 1 (8:30–10:00 a.m.)

All volunteers read and signed the consent form agreeing to participate in the study. After that, a medical questionnaire was administered to collect information on medication use and routine medical visits. Blood pressure measurements were taken after a seated rest for 5 min, and then three recordings were made at 1-min interval on the volunteer’s arm with the highest value. The average of these three measurements was recorded as their BP level. Later, anthropometric measurements were then taken including total body mass, height and waist circumference. Also, the volunteer was asked to fill out the International Physical Activity Questionnaire (IPAQ)—long version.

Finally, the volunteer was sent along with a team researcher to the ergometry laboratory for a stress test. After the stress test, they rested for 15 min and then a blood pressure measurement was taken.

### Visit 2 (8:30 a.m.)

The volunteer (after 12-h fasting) was sent for blood collection (fasting blood glucose, HbA1c, triglycerides, total cholesterol, LDL and HDL cholesterol, creatinine, and glomerular filtration rate). After they snacked, they performed the one-repetition strength test (1RM) consisting of bilateral knee extension, unilateral lying and seated knee flexion, leg press and bilateral plantar flexion.

### Visit 3 (8:00–8:30 a.m.)

The volunteer’s blood pressure was taken and they were sent to the laboratory to perform the assigned intervention (a single session of aerobic exercise OR resistance exercise OR combined exercise). After the exercise session, the volunteer was sent back for blood collection and vascular assessments.

The schedule of visits can be found in the supplementary material. Figure S1 summarizes visit 1 procedure (eligibility), Fig. S2 shows visit 2 procedure and Fig. S3 details visit 3 procedure (intervention day).

### Exercise stress test

The exercise stress test was performed on a treadmill (Inbramed, Porto Alegre, Brazil) using the Bruce Protocol, according the Brazilian Society of Cardiology guidelines^54^. The test was performed under the direct supervision by a cardiologist (blinded for all participants). Peak oxygen consumption (VO_2_peak) was predicted based on total exercise time according to the Bruce protocol^55^ and used only as a cardiopulmonary parameter rather than a control of exercise intensity (see “Exercise protocol” section). Also, factors limiting physical exertion were assessed and estimated aerobic capacity and maximum heart rate (HRmax) were captured (ErgoPC 13, MICROMED, Brasilia Brazil).

### Maximum strength test

We used the one-repetition maximum (1-RM) test to assess strength capacity. Participants were instructed in the proper technique to perform knee extension, knee flexion, leg press and plantar flexion using resistance exercise equipment (Movement Perform W8; São Paulo, Brazil). The warm-up set consisted of 12 repetitions at 30% of the estimated workload for the 1-RM test at a 2:2 (concentric:eccentric) pace determined by an electronic metronome. After a 2-min rest, the 1-RM test started. The test was paused to increase the load when the participant was able to perform more than one repetition and ended when he was not able to complete a full range of motion. A 5-min rest was allowed between retries. The load an individual was able to lift for only one repetition was defined as the maximal workload^46^.

### Exercise protocol

The AE session was performed on a cycle ergometer (Movement BM4500 Pro, São Paulo, Brazil) according to the exercise protocol previously published by our group^46^. It started with 5-min warm-up followed by 40 min at an intensity of 60% of heart rate (HR) reserve monitored by a heart rate monitor (POLAR RS800CX RUN, Helsinki, Finland) and Borg rating scale of subjective perceived effort. We chose to prescribe the intensity of the AE session using HR reserve instead of VO_2_peak because it is a widely recommended method^35,56^ and makes it easy to control intensity during exercise. The VO_2_peak parameter was estimated by the Bruce formula rather than ergospirometry—see “Exercise stress test” section). An exercise duration of 40 min and intensity of 60% of HHR were selected because they seemed a satisfactory dose of exercise^35^ and feasible for sedentary individuals with hypertension (inclusion criteria). The RE session consisted of lower limb exercises (knee extension, knee flexion, leg press and plantar flexion) with 4 sets of 12 repetitions at 60% of 1-RM [2:2 (concentric:eccentric) pace determined by an electronic metronome]. A 90-s rest was allowed between sets and type of exercise. The entire session lasted 40 min. The CE session consisted of a combination of RE followed by AE as described before: RE consisted of only 2 sets of each type of exercise and AE lasted 20 min, totaling 40 min. Participants were not allowed to hold hand rests during lower limb strength exercises; they wore an adjustable seat belt to stabilize their hip while performing knee extension and knee flexion^46^. BP, HR and Borg ratings were assessed at the beginning and every 5 min during the AE session; at the beginning and at the end of the fourth set of each type of exercise during the RE session; and at the beginning of the CE session and then every 5 min of AE and at the end of the second set of each type of exercise (RE).

### Blood flow and flow-mediated dilation

The diameter of the brachial artery and blood flow velocity in the left arm were simultaneously measured by a blinded investigator. These measurements were taken in a quiet, dark room at controlled temperatures (23–24 °C). Participants lay down in the supine position with their arm extended at a ~ 40° angle of the trunk. Following a 20-min rest, baseline BP, resting HR, blood flow velocity and the diameter of the brachial artery were measured (10 min before exercise). They were asked to return to the supine position immediately after the exercise session for BP, blood flow velocity and brachial artery diameter measurements for assessing blood flow, peripheral vascular resistance and endothelial function (10, 40 and 70 min after exercise).

Endothelial function was assessed by FMD of the brachial artery following a technical procedure described in the literature^57^. Participants came to the laboratory in a fasting state; they were instructed to take their medication and refrain from physical exercise. A rapid deflation cuff (Incoterm EC500; Porto Alegre, Brazil) was positioned on the forearm 5 cm distal to the antecubital fossa. Brachial artery B-mode images were taken at the distal third of the arm using a linear multifrequency transducer (12 MHz) attached to a high-resolution Doppler ultrasound machine (Esaote MyLab 70 XVision, Genoa, Italy). The transducer and cuff positions were set to allow repositioning them at the exact same location during testing. The sample volume was adjusted for lumen diameter and data was obtained using an insonation angle of 60°. Baseline diameter scans were recorded over 1 min. The cuff was then inflated to 200 mmHg for 5 min. Image recordings were resumed 20 s before cuff deflation and continued for 3 min thereafter. Real-time Doppler ultrasound video signal was recorded using a USB video card (EasyCAPture; China) and data was stored for offline analysis. Blood flow velocity and brachial artery diameter analyses were performed using edge-detection and wall-tracking software (Cardiovascular Suite Pisa, Italy). Blood flow was calculated at 30 Hz from synchronized blood flow velocity and brachial artery diameter data. Peripheral vascular resistance as calculated as the mean BP divided by blood flow. FMD was calculated as the percentage change in peak diameter following cuff deflation from the preceding baseline diameter. To avoid overestimated FMD values due to low baseline diameter, we used the allometric procedure to scale FMD values [peak diameter/(baseline diameter)^0.89^], as described in the literature^58^ (Table 2). The time to peak diameter was calculated from the point of cuff deflation to the maximum post-deflation diameter^57^. Shear rate (SR) an estimate of shear stress without viscosity (four times mean blood velocity divided by diameter) was calculated and described as the area under the curve (SR-AUC) from cuff deflation to the peak dilation^7^. To assess whether vascular responsiveness actually changes after cuff deflation or is a consequence of lower SR-AUC, we adjusted Δ diameter by SR-AUC [Δ diameter (peak diameter minus baseline diameter) divided by Δ SR-AUC (peak SR-AUC minus baseline SR-AUC)].

### Blood collection

Blood samples were drawn from the participants’ right arm during the ischemia phase of FMD (10 min before and 10 and 70 min after exercise). The first blood sample (4 mL, heparin) was sent for the analysis of parameters of oxidative stress (sulfhydryl, glutathione and carbonyl). To avoid wall cell contamination in further analyses^16^, we changed the syringe and collected an additional 4 mL of blood for EPC (1 mL, EDTA) and EMV detection (3 mL, sodium citrate).

For EPC and EMV analyses, blood samples were processed 4–5 h after the beginning of exercise bout. The analyses of the parameters of oxidative stress were carried out at Hospital de Clínicas de Porto Alegre (HCPA) Thyroid Diseases/Endocrinology Department laboratory and preparation and analyses of EPCs and EMVs were conducted by HCPA Specialized Diagnosis/Laboratory Diagnostic Unit. EPCs and EMVs were assessed 10 min before and 10 and 70 min post-intervention.

### Progenitor cells and endothelial progenitor cells

PCs were immunophenotypically defined^20^ as CD45 + dim/CD34 + and EPCs were defined as CD45 + dim/CD34+/CD309+. Anti-human monoclonal antibodies were added to a 100-μL blood sample at suitable volumes: 3 μL of CD45 PerCP (BD, clone 2D1); 5µL of CD34 PE (BD, clone 8G12); and 5µL of CD309 Alexa Fluor 647 (BD, clone 89,106). Blood samples were incubated protected from light at room temperature for 30 min. Erythrocytes were lysed by addition of 2 mL of 10% BD Pharm Lyse lysing solution (BD Biosciences, San Jose, California) and incubated for 10 min. At least 2 million total events were acquired using a flow cytometer (FACSCanto II) (BD Biosciences, San Jose, California) and analyzed using Infinicyt version 1.7 (Cytognos, Salamanca, Spain). A blinded investigator processed all samples. PCs were calculated as a ratio over CD45 + mononuclear cell counts^16^ by a gating strategy according to the International Society of Hemotherapy and Graf Engineering (ISHGE) protocol^59^. Figure S4 (supplementary material) describes the gating strategy used for identifying CD45 + dim/CD34+/CD309 + cells. PC and EPC were assessed 10 min before and 10 and 70 min post-intervention.

### Endothelial microvesicles

EMVs were immunophenotypically defined as CD45−/CD42b−/CD31 + (31). Blood samples were pre-diluted 1:100 in buffered saline solution (PBS) and 50 μL of the diluted solution were used for cell-labeling reaction. Anti-human monoclonal antibodies with suitable volumes were added to the diluted sample: 5 μL of FITC CD45 (BD, clone HI30), 2 μL of CD31 Alexa Fluor 647 (BD, clone WM59) and 5 μL of CD42b PE (BD, clone HIP1). The samples containing monoclonal antibodies were incubated for 30 min protected from light at room temperature, and 2 mL of PBS was added; they were then centrifuged for 5 min at 540 g. The supernatant was discarded and the pellet resuspended with 500 μL PBS and 50 μL counting beads (BD Biosciences, San Jose, California) were added. A total of one million events were acquired using a flow cytometer (FACSCanto II) (BD Biosciences, San Jose, California) and analyzed using by Infinicyt version 1.7 (Cytognos, Salamanca, Spain). A blinded investigator processed all samples. The amount of EMVs was determined from the number and volume of counting beads. Microvesicle sizes were compared to platelet sizes using average fluorescence intensity of forward scatter, showing a mean diameter ≤ 1.0 μm. Figure S5 (supplementary material) describes the gating strategy for CD45−/CD42b−/CD31 + EMVs. EMV was assessed 10 min before and 10 and 70 min post-intervention.

### Parameters of oxidative stress

All analyses of oxidative stress were consistent with previous studies of our group^60,61^. In short, sulfhydryl content was determined as described by Aksenov and Markesbery^62^, where 5-thio-2-nitrobenzoic acid (TNB) derived from the reaction of thiols with 5,5′-dithiobis (2-nitrobenzoic acid) forms a yellow-colored derivative that is read in a spectrophotometer by measuring the absorbance at 412 nm. The results were expressed in nmol TNB/mg protein. Oxidative stress markers were assessed 10 min before and 10 and 70 min post-intervention.

GSH levels were determined according to the standard method proposed by Teare et al.^63^. Briefly, broteins were precipitated by adding sodium metaphosphoric acid for a final ratio of 1:1. Samples were centrifuged for 10 min at 7,000 g. Fifteen microliters of plasma preparation was incubated with an equal volume of phothaldialdehyde (1 mg/mL methanol) at room temperature for 15 min in the presence of 20 volumes (1:20, v/v) of 100 mM sodium phosphate buffer, pH 8.0, containing 5 mM EDTA. Fluorescence was measured using excitation and emission wave lengths of 350 nm and 420 nm, respectively. A calibration curve was generated using standard GSH (0.001–0.1 mM), and GSH concentrations were calculated as nmol/mg protein.

Oxidized glutathione (GSSG) levels were determined using the enzymatic recycling method^63^, with some modifications. Briefly, plasma was homogenized in 4 (w/v) volumes of a sulfosalicylic acid solution (11%) and Triton X-100 (0.11%) (1:1 ratio). After incubating for 5 min at 4 °C with continuous shaking, the samples were centrifuged at 10,000 g for 10 min (4 °C), and the supernatant was collected for analyses of glutathione levels. For GSSG measurement, 10 μL of the supernatant was added to 110 μL of a GSH masking buffer (100 mM phosphate buffer, 1 mM EDTA, 1.1% 2-vinylpyridine), pH 7.4, and incubated for 1 h at room temperature. The samples prepared for GSSG measurement were subjected to enzymatic analysis in a recycling buffer system containing 300 μM NADPH, 225 μM DTNB, 1.6 U/mL GR and 1.0 mM EDTA in 100 mM phosphate buffer (pH 7.4). The linear increase in absorbance at 405 nm over time was monitored using a microplate reader (SpectraMax M5, Molecular Devices, California, US). A standard curve was generated using known amounts of GSH (100 μM) and GSSG (3.47, 6.95, 13.89 uM).

Duplicate aliquots of plasma (containing ∼0.3 mg of protein) were incubated with 500 μL of 10 mM 2.4-dinitrophenylhydrazine or 1.0 mL of 2 M HCl (blank tube). After 30 min, 250 μL of 50% trichloroacetic acid was added to the aliquots. The samples were subsequently centrifuged at 8000 g for 30 min to obtain the protein pellets, which were immediately washed with ethanol-ethyl acetate at a 1:1 (v/v) ratio. The final protein pellets were resuspended in 500 μL of 8 M urea buffer and incubated at 50 °C for 90 min. The difference between the 2.4-dinitrophenylhydrazine-treated and HCl-treated samples (blank) was used to calculate the carbonyl content determined at 370 nm. Carbonyl content was calculated using the millimolar absorption coefficient of hydrazine (e370 nm = 21.000000 M^−1^ cm^−1^), and the results were expressed in nmol carbonyl/mg protein^64^.

### Statistical analyses

Data were analyzed using SPSS version 24 (Chicago, IL, USA). The results are described as means and standard errors. The Shapiro–Wilk test was used for testing the normality of data and logarithmic transformation was used for variables that were not normally distributed. One-way ANOVA was used to evaluate the characteristics of participants with hypertension at study entry. Furthermore, the generalized estimating equations (GEE) method followed by Bonferroni post-hoc test was used for assessing the effect of each exercise modality (one session of AE, RE or CE) and time point (10 min before and 10, 40 and 70 min post-intervention), and interaction between these two. Cohen's effect size was also applied to FMD measurements (pre- vs. post-exercise session) and classified as non-relevant (0–0.19), small (0.20–0.49), medium (0.50 and 0.79), and large (above 0.80)^65^. *P*-values of less than 0.05 were regarded as statistically significant for any differences.

## Supplementary information

Supplementary information.
