# Supplementary material for: Individuals with controlled hypertension show endothelial integrity following a bout of moderate-intensity exercise: randomized clinical trial
Source: Sci Rep. 2021 Apr 20;11:8528. doi: 10.1038/s41598-021-87990-6 (PMC8058090; doi:10.1038/s41598-021-87990-6)
Supplement: Supplementary file 1 — Supplementary information. [file 41598_2021_87990_MOESM1_ESM.doc]

Original Article

**Individuals with controlled hypertension show endothelial integrity following a bout of moderate-intensity exercise: randomized clinical trial**

**Running title: Endothelium, exercise and hypertension**

Gustavo Waclawovsky^1^, Liliana F.C. Boll^1^, Bruna Eibel^1^, Ana Paula Alegretti^2^, Fabiane Spagnol^2^, Juliana De Paoli^2^, Simone Wajner^3^, Rafael A. Marschner^3^, Maximiliano I. Schaun^1^, Alexandre M. Lehnen^1*^

^1^ Institute of Cardiology of Rio Grande do Sul/University Foundation of Cardiology, Porto Alegre, RS, Brazil.

^2^ Clinical Pathology Laboratory, Hospital de Clínicas de Porto Alegre, Universidade Federal do Rio Grande do Sul, Porto Alegre, RS, Brazil.

^3^ Thyroid Section, Endocrine Division, Hospital de Clínicas de Porto Alegre, Universidade Federal do Rio Grande do Sul, Porto Alegre, RS, Brazil.

* ORCID: https://orcid.org/0000-0002-5912-8020

**Correspondence to:**

Dr Alexandre Machado Lehnen

Instituto de Cardiologia do Rio Grande do Sul

Av. Princesa Isabel, 395 Santana, 90620-001 Porto Alegre – RS Brazil

Phone:+55(51)32303600, branch 3636/3757 – Fax:+55(51)32303600 branch 3757

[amlehnen@gmail.com](mailto:amlehnen@gmail.com)

**Laboratory of Clinical Investigation**

**- IPAQ – long version**

**- Volunteer sent for stress test at Institute of Cardiology of Rio Grande do Sul.**

Lasts: ~25 min

**Laboratory of Clinical Investigation**

**- SBP/DBP* and HR measurements**

Seated (5 min), 3 recordings at 1-min interval on the arm with highest values.

*****If SBP ≥160 mmHg and/or DBP ≥105 mmHg, referred to a medical provider and a new visit is scheduled.

Lasts: ~10 min

**Laboratory of Clinical Investigation**

**- Medical questionnaire:**

Personal information

Medical visits

Medication use

Drug compliance

Regular diet and exercise

Other health conditions

Lasts: ~15 min

**Laboratory of Clinical Investigation**

**- Meeting eligibility criteria confirmed**

**- Team explains the study**

**- Consent form signed if they agree to participate**

Lasts: ~15 min

**Volunteer arrives at LCI**

**8:30 – 10:00 a.m.**

**(appointment pre-scheduled via phone call)**

**Figure S1 – Volunteers’ eligibility (visit 1)**

**Laboratory of Clinical Investigation**

**- Anthropometric measurements**

Total body mass

Height

Waist circumference

Lasts: ~10 min

**Institute of Cardiology of Rio Grande do Sul**

**- Stress test performed by a cardiologist with researcher’s attendance**

Test preparation

Stress test

Post-test recovery time

Lasts: ~35 min

**End of visit 1**

**10:30 – 11:50 a.m.**

**Visit 2 scheduled**

**Step 1**

**Step 2**

**Step 3**

**Step 4**

**Step 5**

**Step 6**

**Figure S2 – Biochemical and physical assessments (visit 2)**

**Laboratory of Clinical Investigation**

**- Regular snack and rest for 45-60 minutes**

**- 1RM test**

Bilateral knee extension

Unilateral lying and seated knee flexion

Leg press

Bilateral plantar flexion

**- Rest for 15 minutes**

**- SBP/DBP* and HR measurements**

Seated (5 min), 3 recordings at 1-min interval on the arm with highest value.

***** Volunteer is released when BP is back to baseline values.

Lasts: ~1h30

**Laboratory of Clinical Investigation**

**- Blood collection**

Fasting blood glucose

HbA1c

Triglycerides

Total cholesterol

HDL cholesterol

LDL cholesterol

Creatinina

GFR

Lasts: ~15 min

**Laboratory of Clinical Investigation**

**- Visit checklist**

Fasting (12h) confirmed

Procedure explained

Medication use checked

Questionnaire administered to collect information on last meal time and content

**- SBP/DBP* and HR measurements**

Seated (5 min), 3 recordings at 1-min interval on the arm with highest value.

***** If SBP ≥160 mmHg and/or DBP ≥105 mmHg, refer to a medical doctor or nurse; a new visit is scheduled.

**- Volunteer is sent for blood collection.**

Lasts: ~30 min

**Volunteer arrives at LCI**

**8:30 a.m.**

**(appointment confirmed on a phone call two days in advance)**

**End of visit 2**

**10:30 – 10:45 a.m.**

**Visit 3 scheduled**

**Step 1**

**Step 2**

**Step 3**

**- Aerobic exercise session (40 min)**

Horizontal cycle ergometer

60% HR reserve

Borg’s scale: score 12–13

**- Strength exercise session (40 min)**

4 types of exercise (lower limbs)

4 sets x 12 repetitions

60% 1RM;

90-second interval between sets and types of exercise

Concentric and eccentric phases (2/2 cadence).

**- Combined exercise session (40 min)**

**Strength exercise (20 min):**

4 types of exercise (lower limbs)

2 sets x 12 repetitions

60% 1RM

60–90 second interval between sets and exercise modality

Concentric and eccentric phase (2/2 cadence)

**Aerobic exercise (20 min):**

Horizontal cycle ergometer

60% HR reserve

Borg’s scale: score 12–13

(*) Visit terminated and new appointment scheduled

SBP ≥160 mmHg

DBP ≥105 mmHg

**- Endothelial function assessed by FMD**

10 minutes after exercise

40 minutes after exercise

70 minutes after exercise

**- Blood collection**

10 and 70 minutes before exercise

4 mL (heparin) – oxidative stress

1 mL (EDTA) – CPs and ECPs

3 mL (sodium citrate) – EMVs

Lasts: ~1 hour and 30 minutes

**- Assigned exercise intervention performed**

Session duration: 40 minutes

SBP/DBP: monitored every 5 minutes during aerobic exercise session and at the end of the last set of strength exercise

**- Endothelial function assessed by FMD**

10 minutes before exercise intervention

**- Blood collection**

10 minutes before exercise

4 mL (heparin) – oxidative stress

1 mL (EDTA) – CPs and ECPs

3 mL (sodium citrate) – EMV

Lasts: ~30 minutes

**- SBP/DBP* and HR measurements**

Seated (5 min), 3 recordings at 1-min interval on the arm with highest value.

**- Volunteer asked about last meal**

Night before the intervention

Lasts: ~10 minutes

**Volunteer arrives at LCI**

**8:00 – 8:30 a.m.**

**Fasting ≥8h**

**(overnight fasting)**

**Figure S3 – Data collection on intervention day (visit 3)**

**
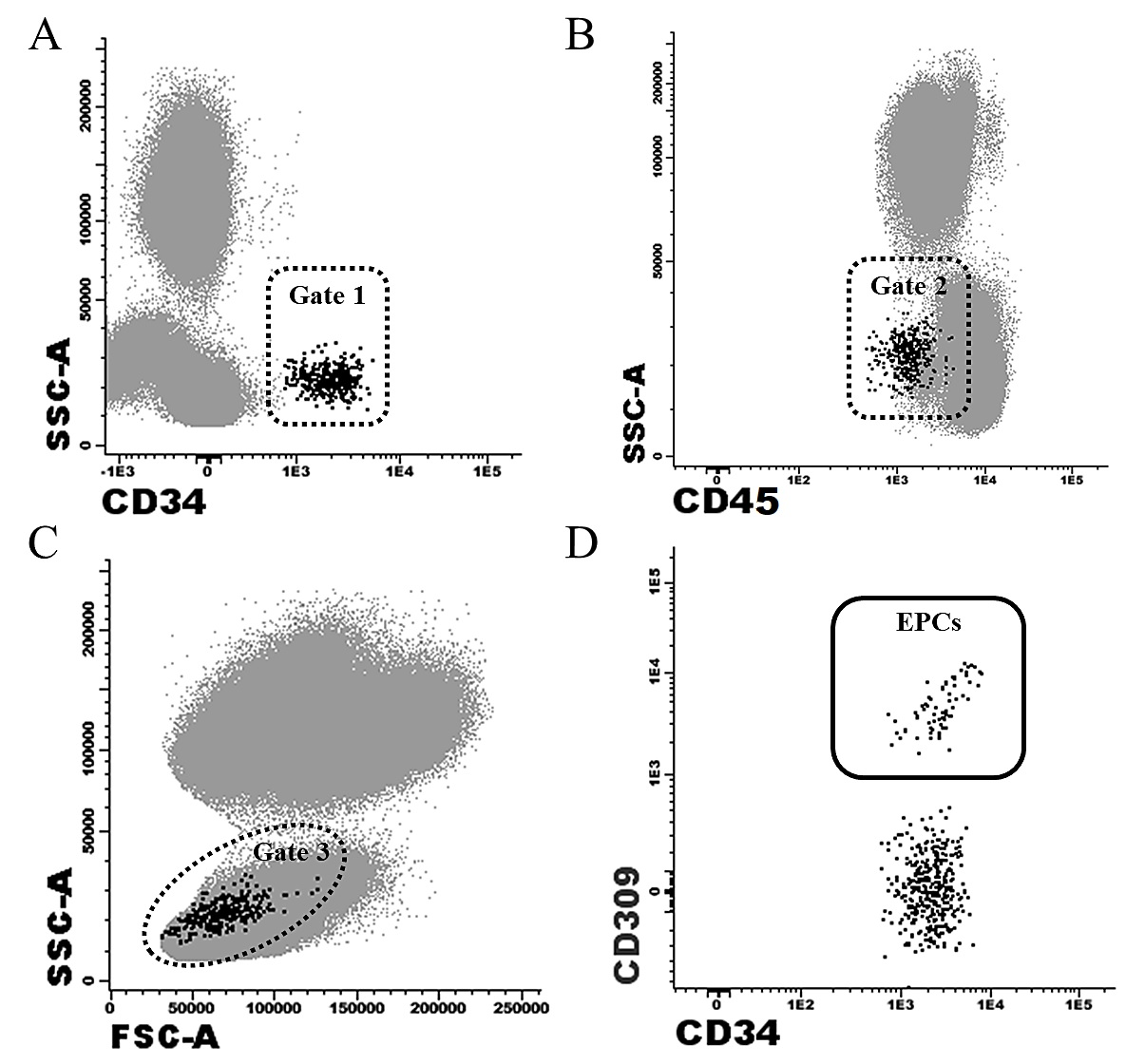
**

**Figure S4 – Analysis of CD45-PerCP, CD34-PE and CD309-Alexa Fluor 647-labeled peripheral blood cells.** A: (Gate 1) Selection of CD34+ cells; B: CD45 versus side scatter (SSC) to define CD45+ cell population by identifying CD34+ cells in CD45+dim (Gate 2); C: Selection of PC cell population using forward scatter (FSC) versus SSC located in the mononuclear region (Gate 3). D: Identification of CD309+ cell population from total progenitor cells representing endothelial progenitor cells (EPCs) (CD45+dim/CD34+/CD309+).

**
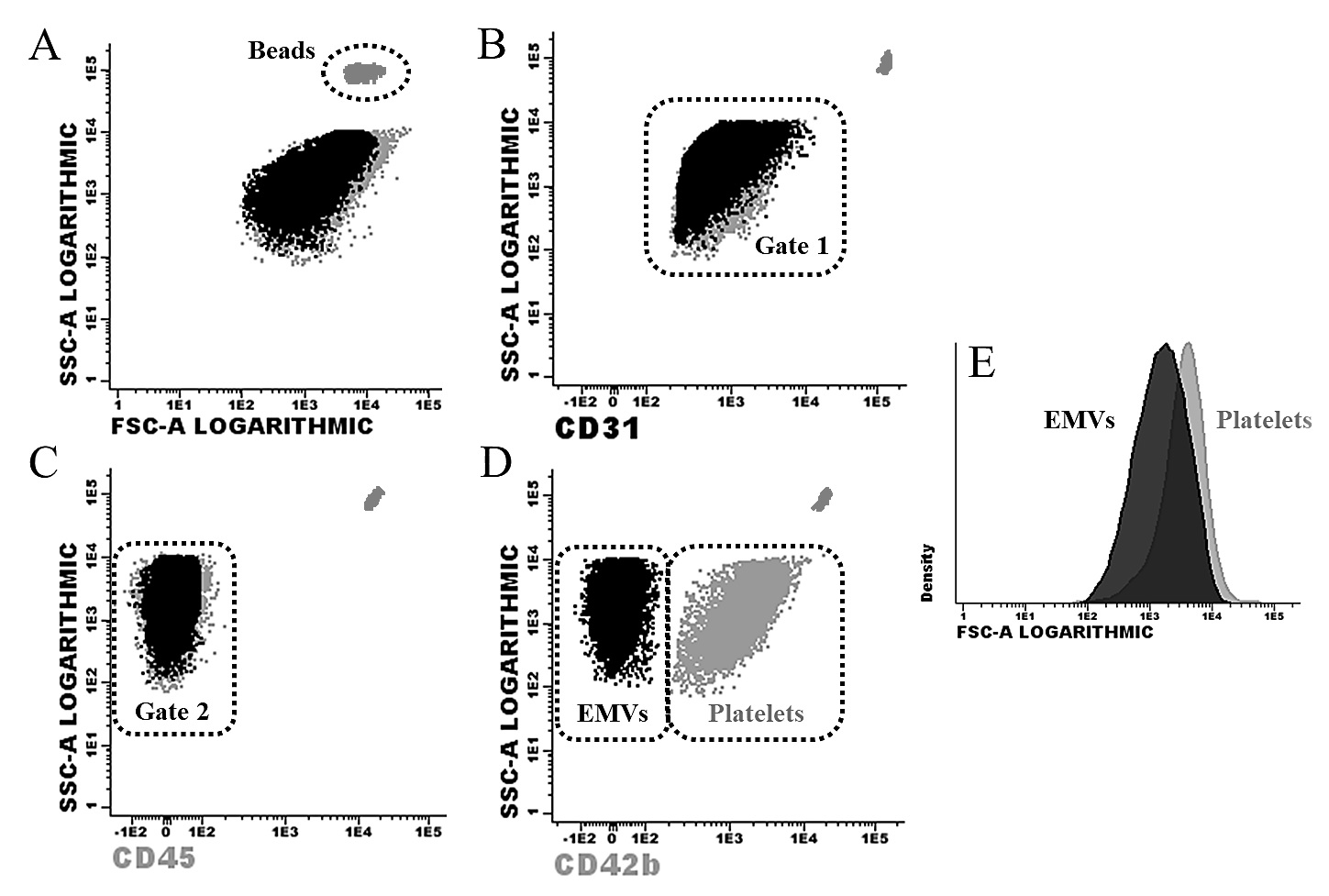
**

**Figure S5 – Plots representing the analysis of endothelial microvesicles (EMVs) by flow cytometry in whole blood samples from hypertensive participants.** A: Total events after acquisition on a logarithmic scale for selecting events that are smaller than leukocytes using beads for quantifying EMVs. B: Gate 1 selecting positive events for CD31-Alexa Fluor 647 marker that is expressed in both EMVs and platelets. C: Selection of all CD31+ events that are CD45– (Gate 2). D: CD42b marker used for differentiating EMVs (CD45–/CD42b–/CD31+) from platelets (CD45–/CD42b+/CD31+). D-E: Histogram showing that EMVs are smaller than platelets (mean ≤ 1 μm).


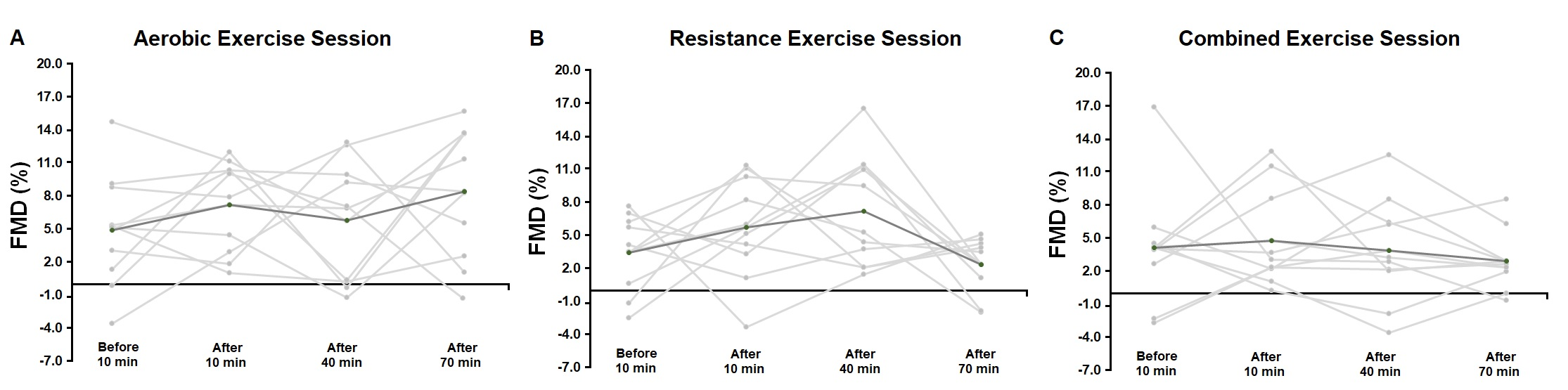


**Figure S6 – Plot individual data points for flow-mediated dilation (FMD).** The dark-gray line represents the mean of group.

**Formulas used in the study**

1. LDL cholesterol = (total cholesterol – HDL cholesterol) – (triglycerides / 5);
2. GFR (mL/min x 1.73 m^2^) = 175 x creatinine – 1.154 x age – 0.203 x [1.212 (for black population) or 0.742 (for females)];
3. FMD (%): (peak brachial artery diameter following cuff deflation * 100) / (mean resting brachial artery diameter) – 100;
4. Mean blood flow velocity (cm/s): anterograde velocity – retrograde velocity;
5. Blood flow (mL/min): (blood flow velocity * area) * 60;
6. Shear stress rate/second: 4 * (blood flow velocity / artery diameter);
7. Vascular resistance (unit): mean blood pressure / blood flow.
